# Supplementary material for: A Humanized Yeast Phenomic Model of Deoxycytidine Kinase to Predict Genetic Buffering of Nucleoside Analog Cytotoxicity
Source: Genes (Basel). 2019 Sep 30;10(10):770. doi: 10.3390/genes10100770 (PMC6826991; doi:10.3390/genes10100770)
Supplement: Supplementary file 1 [file genes-10-00770-s001.zip › Additional_Files/Additional_File7_GOTermHeatmaps.zip!/Additional_File7_GOTermHeatmaps/oxidoreductase_activity,_acting_on_paired_donors,_with_oxidation_of_a_pair_of_donors_resulting_in_the_reduction_of_molecular_oxygen_to_two_molecules_of_water.pdf]

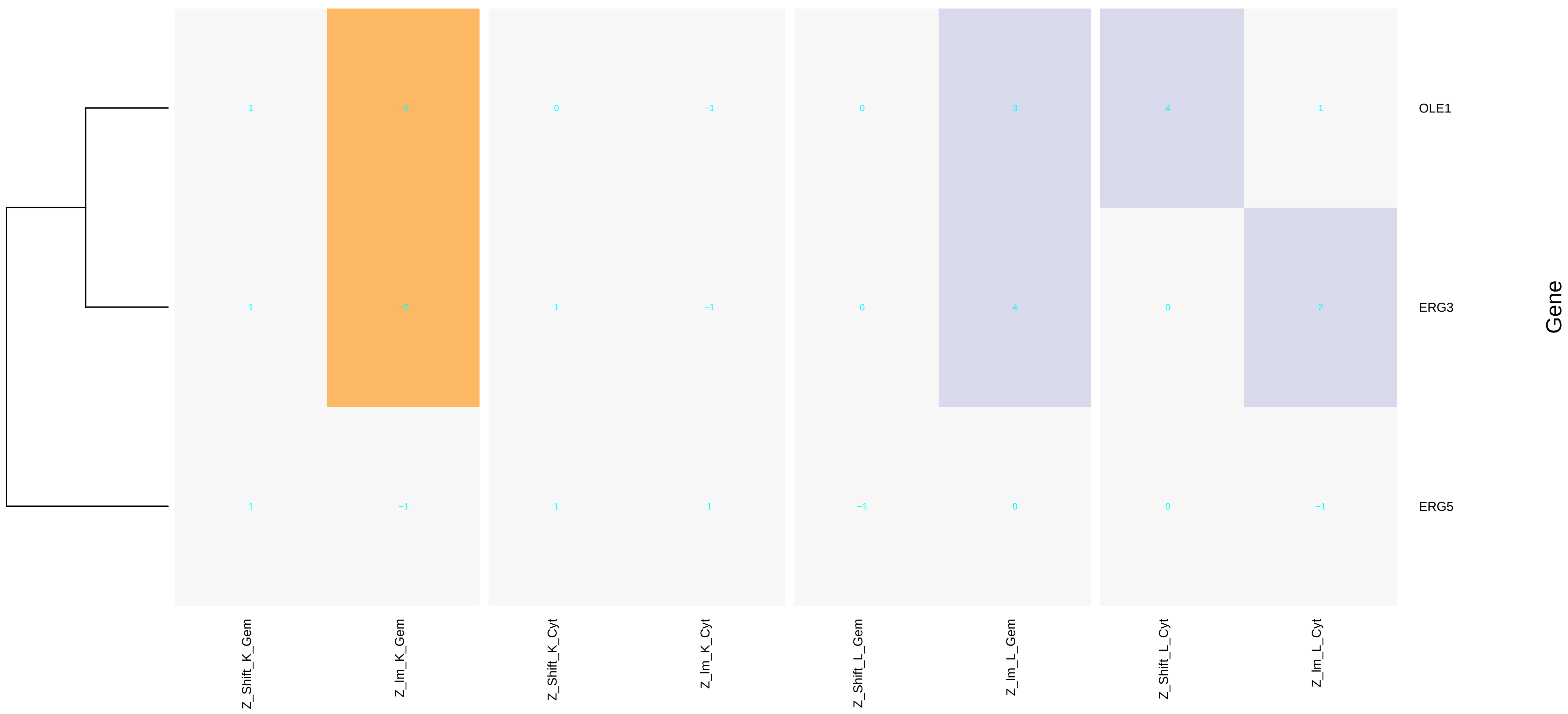

1
−5
0
−1
0
3
4
1
OLE1
1
−5
1
−1
0
4
0
2
ERG3
1
−1
1
1
−1
0
0
−1
ERG5
Gene
Z_Shift_K_Gem
Z_Im_K_Gem
Z_Shift_K_Cyt
Z_Im_K_Cyt
Z_Shift_L_Gem
Z_Im_L_Gem
Z_Shift_L_Cyt
Z_Im_L_Cyt
